# Interactions with Home and Health Environments Discourage Physical Activity: Reports from Children with Complex Congenital Heart Disease and Their Parents

**DOI:** 10.3390/ijerph18094903

**Published:** 2021-05-04

**Authors:** Patricia E. Longmuir, Mary Corey, Brian W. McCrindle

**Affiliations:** 1 Labatt Family Heart Centre, The Hospital for Sick Children, Toronto, ON M5G 1X8, Canada; brian.mccrindle@sickkids.ca; 2 Child Health Evaluative Sciences, The Hospital for Sick Children and Dalla Lana School of Public Health, University of Toronto, Toronto, ON M5G 1X8, Canada; mary.corey@sickkids.ca; 3 Department of Paediatrics, Faculty of Medicine, University of Toronto, Toronto, ON M5G 1X8, Canada

**Keywords:** inactive, congenital heart disease environment, school environment, parent environment, peer environment, children with complex congenital heart disease

## Abstract

Children with complex congenital heart disease are less active than recommended for optimal health, with social and physical environments important determinants. The purpose of this study was to examine the physical activity perceptions of children with complex congenital heart disease and their parents to identify social and physical environment intervention targets. A semi-structured discussion guide elicited physical activity perceptions from children (26 boys, 19 girls, 6.0–12.4 years) with complex congenital heart disease (single ventricle *n* = 42) and their parents during three child and three parent focus groups and 41 interviews. Interviews and focus groups were audio-recorded and transcribed verbatim for inductive thematic analysis. Children and parents identified home, peer and health environments as impacting on their children’s physical activity participation. Peer environments, such as school or daycare, were supportive by providing physical activity facilities and enabling fun with peers and time outdoors. At home, parent and sibling interactions both encouraged and discouraged physical activity. The children’s unique health environment fostered physical activity uncertainty, discouraging activity despite minimal or no physician recommendations to restrict physical activity. Children with complex congenital heart disease and their parents recognize the importance of physical activity and fun with friends. Physical activity uncertainty contributes to their inactive lifestyles despite minimal restrictions from health professionals. Positive clinical encouragement and health environment interventions that better support physical activity are required.

## 1. Introduction

Physical activity is critically important for children’s physical and psychosocial health, both during childhood and for the rest of their lives [1,2,3,4]. Children with complex congenital heart disease (CCHD) are less active than their peers [5,6,7,8], even at a young age [9]. Exercise training can improve the fitness of these children [10,11,12], with benefits maintained over the short [13] and long term [14]. However, their physical activity participation is lower, and unrelated to their exercise capacity [7], even when exercise capacity is near-normal [8]. Studies consistently indicate that a small proportion of these children are able to achieve the physically active lifestyles associated with optimal health [15,16]. Taken together, these findings suggest the need to examine factors other than the physiological effects of the CCHD as important determinants of their physical activity behaviour.

Social and physical environments combine with individual characteristics and behaviours as important determinants of health [17]. Previous investigations among children with CCHD have focused on correlates between physical activity and individual characteristics, primarily those related to the diagnosis [18,19,20], with little attention having been paid to environmental influences. Abnormal CCHD physiology limits exercise capacity but the degree of limitation is not associated with activity participation [18]. Although exercise is safe for most children with CCHD, 20% report physician-imposed activity restrictions [18]. Teens with CCHD recognized the importance of physical activity for health, but interpreted feelings of fatigue and limited activity self-efficacy as negatively impacting on their participation [19]. Interactions with a supportive social environment and mastery experiences were perceived as facilitating physical activity [19]. These findings align with theories that describe how behaviour is influenced by social interactions and interpersonal environments [21]. Interactions that support the individual’s autonomy (e.g., mastery experiences) will increase the desired behaviour while those that are controlling (e.g., authoritarian style) will decrease motivation and enjoyment. These theories would posit that the importance of personal attitudes and beliefs toward the behaviour (e.g., physical activity is important for health), the interpretation of social norms about the behaviour (e.g., supportive social environment), and the perception of self-control over decision-making and action (e.g., activity self-efficacy) would impact engagement in physical activity behaviours.

That 80% of parents of children with CCHD underestimate their children’s exercise capacity [22] and 20% report activity restrictions not required by the child’s physician [23] has fostered a narrative emphasizing the potential role of overprotection [24,25] by parents, teachers or other adults [18]. Healthcare professionals have also identified concerns about overprotection [26]. These data suggest that, in contrast to healthy children [27], the social environment experienced by children with CCHD may be interpreted as more likely limiting rather than promoting physical activity. The impact of the physical environment, such as schools and community spaces [28], on the physical activity of children with CCHD has not been investigated. The aim of this retrospective study was to understand how children with CCHD and their parents interpret the physical activity interactions they experience within social and physical environments. 

## 2. Methods

### 2.1 Research Approach

The physical activity perceptions of children with CCHD and their parents were investigated using an approach that we modified from the interpretive interactionist foundation originally described by Denzin et al. [29]. In this study, interpretation was used to enable participants and researchers to investigate how experiences were understood and why behaviour changed as a result [30]. Interactionism emphasized current behaviour would be impacted by important “life-changing” experiences, such as the treatment of CCHD; those experiences would both change individuals and be changed by them. Grounding this research in a constructivist framework allowed physical activity behaviours of children with CCHD to be changed over time through their interaction with multiple factors, experiences and settings. 

### 2.2. Study Design

This retrospective qualitative research study analyzed data collected from children with CCHD and their parents using both focus group and interview methods. The data were collected during baseline assessments for children enrolled in a randomized controlled trial to encourage physically active lifestyles [31]. Separate child and parent focus groups or interviews were conducted using semi-structured discussion guides. 

### 2.3. Participants

Children, 5 to 12 years of age, with CCHD and their parents were invited to participate in a focus group and/or interview. The responsible cardiologist introduced the study. The research team provided additional information and determined participation. Written informed consent or assent was obtained from the parents and children, respectively.

Participants were 26 boys (mean age 9.0 ± 1.6 years) and 19 girls (mean age 8.9 ± 1.9 years), most (*n* = 42) with single ventricle physiology, and their parents. Four children (2 boys, 2 girls, age range 8.7 to 11.5 years) completed only a focus group discussion. They attended with 4 mothers and 2 fathers from 5 families. Of the 41 interview participants, 6 boys (age range 8.4 to 12.4 years) and 5 girls (age range 6.5 to 11.5 years) also participated in focus group discussions. Purposive [32] sampling was employed as a maximum variation strategy [33] to include those with one/multiple surgeries, CCHD treatment in infancy/early childhood, reparative/palliative surgeries, and right/left side CCHD. Demographic information for the focus group participants is provided in Table 1 and for the interview participants in Table 2. Interview participants were representative of the population of children who have the Fontan procedure for single ventricle, except that girls were more represented by the sex-stratified recruitment strategy [34].

### 2.4. Focus Groups and Interviews

Separate parent (*n* = 3) and child focus groups (*n* = 3), 4–6 participants per group, facilitated the expression of personal opinions. One child (D) would not separate from his parents. His father was present during his child focus group and he was present during his parents’ focus group. Given their age, most children were interviewed with a parent seated nearby. Parents received a lengthy questionnaire to minimize their attention to and involvement in the children’s responses. Parent interviews were conducted while the child played or completed study assessments.

Each discussion began with the researcher reminding participants that their participation was voluntary and confidential. It was also emphasized that what was said would not be shared with the healthcare team in a way that participants could be identified. Interviews and focus groups with children utilized art and craft activities to build rapport and enable children to express their thoughts in ways other than verbal description. Each discussion began with general questions about the children’s leisure interests to encourage the development of rapport between the researcher and discussion participants. The semi-structured discussion guide was then used to investigate key points while allowing new themes/issues to emerge. Questions explored home/ school/community/peer activities, physical activity guidance from medical professionals and others, and perceptions of new or difficult activities. Parents were also asked whether Health Canada’s physical activity recommendations [35] were appropriate for children with CCHD, about their children’s physical activity barriers, and about their activity expectations. All discussions were audio-recorded. A research assistant documented parental influence, non-verbal communication and features of the discussion environment (e.g., interruptions, distractions).

### 2.5. Data Analyses

Audio recordings were transcribed verbatim using pre-determined transcription conventions. Data management used NVivo 12 software (QSR International, 2018, Burlington, MA, USA). Assigned identification codes (“boy” or “girl” with consecutive numbers for interviews or letters for focus group only) replaced all identifiable information. Parent pseudonyms were “mom” or “dad” followed by the number or letter for the child (e.g., MomA was the mother of GirlA). Focus groups were 60–90 min. Interviews were 30 (children) to 60 min (parents). Coding and inductive analyses were done by the first author and reviewed with the study team. Content familiarity was maximized through multiple transcript reviews while listening to the audio recordings. Each focus group/interview was coded separately, in the order of completion. Inductive analyses began with key phrase identification. Initial codes were created to identify significant concepts. Inductive and analyst-constructed typologies [33] organized the results. Analyst-constructed typologies were physical activity at home/school, activity preferences and perceptions of child/parents, medical influences (cardiologist, CCHD), and children’s physical activity supports and barriers. Examples of inductive typologies expressed by participants were having fun and uncertain expectations. 

The final analytical phase interpreted the results relative to understanding why children with CCHD are less physically active. A situational map was created to represent the identified themes. Children’s physical activity was placed in the centre and identified concepts added using a plus (+) symbol for encouraging and a minus (−) symbol for discouraging influences. Finally, concepts were reviewed and grouped in related environments (home, health, peer).

## 3. Results

**Figure 1:**
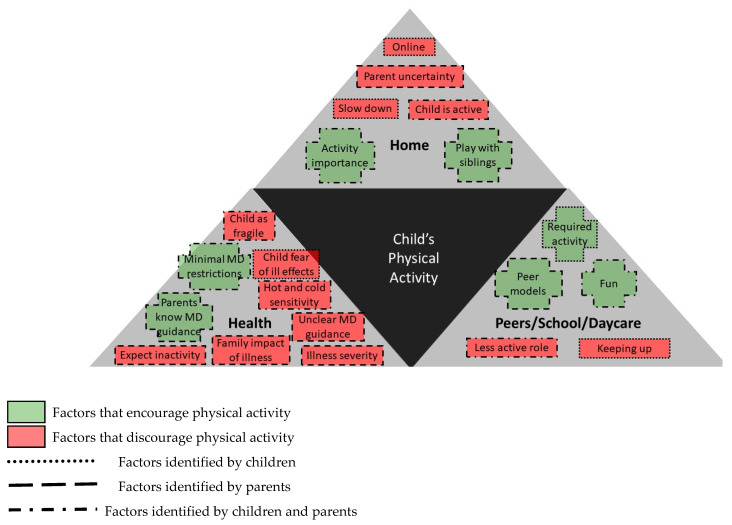
Situational Map of Environments Impacting on the Physical Activity of Children with Complex Congenital Heart Disease.

Children with CCHD and their parents reported interactions within three environments as impacting on the children’s physical activity participation (Figure 1). The social environment at home identified parent and sibling interactions as key influencers. The social health environment encompassed hospital and healthcare team experiences, factors uniquely important for these families. The third environment emphasized supportive social and physical environments related to peer interactions at school/daycare.

### 3.1. Peer Physical and Social Environments (School/Daycare) Support Physical Activity

Peer environments (e.g., school, daycare) were interpreted as enhancing physical activity participation even though children recognized the impact of personal characteristics (e.g., self-efficacy) in relation to “keeping up” with peers. Children’s favourite activities were with peers (e.g., swimming, biking, dancing, tobogganing, or building snowmen). Boys and older girls also mentioned sports (e.g., basketball, volleyball, baseball, soccer). Children recognized that their limited skill and/or endurance discouraged their activity with peers (*GirlA: I don’t really like to be active, cause sometimes I get tired out. I mostly watch TV...).* Encouragement from peers and having fun were most important. Boy30 enthusiastically spoke of his friends and how they all played on baseball teams in the same league. Most children indicated that, when by themselves, they usually watch TV or play video/computer games (*Boy20: At home, I watch a lot of TV but I get to my work first and then I go to TV).* None of the children indicated they would play video/computer games when with friends. 

Although interactions within peer environments were reported as encouraging activity, children with CCHD often described themselves as adopting more sedentary roles. They played goal or only did batting in baseball. Boy3 said he liked tennis but later indicated that he liked watching tennis but had never played. Another child (Girl17) said she played in a neighbour’s pool but was adamant she didn’t swim. 

Parents emphasized their children’s activity at school, during recess or physical education class, and the mandatory daily physical activity requirement. Children reported school/recess/physical education activities focused on team sports (e.g., soccer baseball, dodgeball). After-school childcare settings were also described as active (*Girl10: I go to my babysitter’s house, and we play hide ‘n’ seek).* Children perceived these activities as required rather than for enjoyment (*Boy7: We play soccer and play basketball at 6:30).* Since only 12 of 45 children indicated they went to daycare (*n* = 5) or a babysitter (*n* = 7) after school, most children with CCHD do not have access to these after-school peer opportunities.

### 3.2. The Social Home Environment Encourages and Discourages Activity

The home environment was interpreted as both encouraging and discouraging children’s physical activity. Children and parents recognized the importance of activity for heart health and thought they had achieved a physically active lifestyle despite objective measures indicating only 28% achieved the recommended 60 min per day [35]. Siblings were perceived as having a positive impact (e.g., Mom5 indicated her child with CCHD had always been very cautious but became much more active when playing with his younger brother). The children’s participation in a variety of home-based activities (e.g., soccer, swimming, biking) was described by parents and children (*Boy11: I sometimes go rollerblading and I scooter and I swim sometimes*). The physical home environment was also important. Having opportunities for active play interactions at or close to home definitely seemed to encourage more activity, in both summer and winter:

*Girl10: ‘Cause there’s a hill … in the wintertime there’s snow and we can slide down. And in the summertime we go to a really close park.*

Children reported that their time at home was often spent using screens, watching videos, being on social media or playing video games. *Girl7*: *When I get home from school, I do homework. Go on the computer. I like to watch T.V.* The growing interest in screens was perceived as limiting the children’s activity.

Parents recognized that keeping their children active was often difficult, despite its importance. Several parents spoke about their children’s preference for sedentary activities. In enrolling her daughter in the physical activity study, Mom13 was seeking support so that her daughter would initiate her own physical activity.

*Mom13: I want her to think of physical activity first rather than second. Generally, we have to encourage her to be physically active. … She would rather watch TV or play computer games, etc. But when she’s being active, she enjoys it, it is just the second thought, not the first thought.*

Contrary to a narrative of adult overprotection, parents perceived that, at times, it was their children who resisted physical activity. One mother (of Girl6), a physical education teacher, used her expertise to find alternate activities when her daughter became concerned that being active might be too much for her heart. Dad5 described the panic that his son experienced if he was pushed into physical activity:

*Dad5: Convincing him that it’s a good thing to do. … When he’s comfortable that’s when he engages. If you try to force him, it’s impossible. He freaks out.*

Parents also recognized their own challenges with fully supporting an active lifestyle for their children. A father (of Girl2) indicated his wife did not enjoy physical activity and was content to have the children inside the house. Another parent (Dad9) emphasized the need to encourage other family members to participate. Many parents indicated they had concerns about the child being injured if they sustained a blow to the chest. Children also were aware of their parents’ concerns about getting hit in the chest during activity.

*Boy20: I really wanted to take baseball but it’s “No”, my Mom and Dad said, because um, like, in case the ball comes towards me and it hits my chest.*

Parents also reported limiting children’s activities when they perceived that their child might not be successful in a particular activity. Mom6 indicated they had steered her daughter to softball and volleyball, as they felt she could be successful. She said, *I think we do subconsciously eliminate a lot of things that maybe she’d like to try and, even though she may not have tremendous success, she might enjoy it.*

Interestingly, all of the children commented on what their parents said to them regarding physical activity. One child, who described herself as not liking to be active, talked about how much her mother wanted her to be more active. In contrast, all of the other children spoke about their parents limiting their activity. Children said they were often told to settle down or sit still. Parents recognized they would limit children’s participation when feeling uncertain about their physical activity capacity. These limits were frustrating for children wanting to be active:

*Mom3: His father is like, “oh, I am scared”.*

*Boy3: Nothing will happen. I play soccer every day and I’m good at it. I got nine goals.*

*Researcher: So, did you play on a soccer team this summer?*

*Boy3: [shakes head] My dad and, uh, like when we play soccer, it’s rough. … But I just keep passing. Two persons coming and I just go through them and then they bump into each other.*

Despite their frustration, children seemed to have internalized parental guidance as indicated by the advice they would give to other children with CCHD.

*Girl8: Just keep trying and if you need a break just slow down or stop.*

*Boy1: I think you need to be careful sometimes, like me.*

*Boy6: Run about a 100m and then stop … and then run again.*

### 3.3. Health Environment Interactions were Interpreted as Discouraging Physical Activity 

Most children could not recall talking with a doctor about physical activity. Discussions they did recall were perceived as limiting activity. One child (Boy20) said his doctor wanted him to “*be active sometimes*”. Another (Girl10) remembered being told to be active “*just a little bit*”. A third (Boy13) indicated he could not go “*upside-down*”. Since children determine their own activity at school and with peers, the limited information they could recall was surprising. Of concern, two children who were certain their doctor had never spoken to them about physical activity would actually have specific restrictions (due to a pacemaker or risk of sudden death during isometric exercise).

Both parents and children recognized their CCHD made it difficult to tolerate hot or cold environments, physical parameters affecting their ability to be outdoors. For example, Mom6 reported that her daughter enjoys swimming in the summer but quickly gets “*really cold*” in an indoor pool.

Parents could easily recite the physical activity guidance conveyed by their children’s cardiologists, and that few if any restrictions were required. Parents of children with CCHD affecting the left side of the heart reported isometric activity restrictions (*MomD: No, they’ve given us no restrictions except not lifting weights).* Children with implanted devices were to avoid being hit in the area of the device or leads (*Mom C: Anything that could put risk to getting hit where his pacemaker is, but that’s really about it*). One mother uniquely recounted how her child’s cardiologist counselled her to be confident that her child’s body would regulate play activities to an appropriate intensity.

*Mom A: We were told by [cardiologist] that if she is physically not capable of doing something her body will just naturally slow down or suggest that she sit down and rest. She won’t have a choice. So, she can go as hard as she wants, as much as she wants. That body itself will let her know if it’s had too much, but no limitations.*

Parents interpreted current and past CCHD experiences as discouraging rather than encouraging physical activity. Despite knowing the cardiologists’ physical activity recommendations, parents reported often feeling uncertain about what was appropriate physical activity for their children. Even the parents of a boy who played ice hockey with body checking expressed concern about the possibility of him being hit in the chest (Dad10). Expectations from healthcare professionals that their children would be relatively inactive increased parental uncertainty when children were perceived to lead active lives.

*Dad D: At Sick Kids, they said he’s going to be lethargic. They said he’s going to be tired all the time or like …*

*Dad B: Exhausted.*

*Mom D: It hasn’t happened. It never happened.*

Parent uncertainty was also linked to the child showing signs of exertion. One parent described her difficult “parenting moments” in relation to her child’s concern about being too active:

*Mom A: If she says, “I can’t do something ‘cause I’m tired” or, you know, she’ll put her hand to her chest and, of course, I immediately think, ‘oh, so here’s one of those parenting moments. Do I force her to do it ‘cause I think she’s playing with me or, you know, or do you allow her that opportunity or not to let her do it?’.*

One common story among all parents was the high-stress experience of having a critically ill young child. Although all of their children had successfully come through that difficult period, it was clear those experiences, and the ongoing health risks their children face, continue to influence parents’ perceptions of their children’s capacity for physical activity. Parents struggle to balance their responsibility to encourage physical activity with their own fears in relation to their children’s fragile health. Parents recognized the importance of avoiding overprotection (*Mom A: Even though it’s hard …You try not to hermetically seal them in a bubble)* but were uncertain as to how to distinguish the effects of exercise from symptoms of heart problems (e.g., sweating, increased heart rate). They described separation anxiety as the children’s health allowed more vigorous activity.

*Mom E: I have to remember when he’s running, he’s going to sweat. Profusely, potentially, if that should be just his natural thing. But then I get scared because then I see the sweating and then I have to [chuckle] remind myself that his heart rate will increase ‘cause he’s physically active. But I’m always going there and I’m checking it and, you know, seeing what his heart rate is and things. … I have to remind myself there’s all those normal things, but it’s always in the back of my mind … So, it is very anxious for me. And I think I’m growing out of it ‘cause you can see by his scraped knees and stuff that [chuckles] he is getting active. But I’m having separation anxiety between the role of constantly mother hen-ing over him and realizing that exercise is good and I have to allow him to do that. So, it’s a balancing act for me.*

It was also clear that concerns over the children’s health extend well beyond family members to other adults in the children’s lives, such as teachers, daycare staff and parents of friends.

*Mom A: She just reached an age where sleepovers are happening. A couple of parents have said, “Do I need to know anything? If anything were to happen what do I do?”*

## 4. Discussion

Creating activity supportive social and physical environments is important to children’s participation [36]. Among adults, long-term physical activity engagement is linked to intrinsic motivation, whereas more self-identified priorities (e.g., health benefits) contribute to the initiation of new activity habits [37]. Competence for activity is also critically important. Young adults with CCHD have reported feelings of uncertainty, being different, and a lack of knowledge about the impact of their diagnosis, which was in part attributed to their exclusion from discussions between their parents and healthcare providers. Although it is recognized that the activity environments differ between children (e.g., school-based activities, lessons) and adults [37], the importance of motivation and competence have also been recognized in relation to the physical activity of adolescents with CCHD [19,21]. Children in this study were 6 to 12 years of age and thus differed in relation to their autonomy for physical activity participation and their capacity to interpret their experiences and interactions with their social environment. Nevertheless, they reported that competence (i.e., keeping up with peers), social norms (i.e., feedback from parents and others) and the perception of self-control over decision-making and action (e.g., activity uncertainty) impact their engagement in physical activity.

Children with CCHD are predisposed to inactive lifestyles in the same way their peers get insufficient activity for optimal health [38], with family/home and school/community environments being influential [36]. In addition, the physical activity of children with CCHD is influenced by their health environment. That is, their experiences with their CCHD and their interactions with healthcare professionals and settings contribute to their interpretations of physical activity interactions. Despite concerns about being able to “keep up”, children with CCHD and their parents reported peer environments as primarily supportive, with fun with friends being very important to the children’s physical activity. At this age, peers encouraged, and school/daycare environments facilitated, participation in active games/sports. Home environments were reported to both encourage and discourage physical activity. Participants recognized the importance of active lifestyles for health and siblings were a significant motivator. Nevertheless, children reported their interactions at home to be primarily inactive; using screens or parents who often asked them to “sit still” or “slow down”. Interactions within the health environment were interpreted as increasing activity uncertainty, despite parents’ knowledge that physical activity restrictions for their children were minimal. Physician guidance was unequivocal but also unclear, particularly when children’s observed activity differed from expectations established in the health environment. Rather than a narrative of overprotection [19], parents identified a careful “balancing act” between encouraging healthy active lifestyles and respecting their interpretation of health environment interactions that emphasized monitoring their children for signs of heart problems. Some children were also hesitant to be active because it “might be too much for my heart” or they might be injured and have to go to the hospital.

Being encouraged by significant others and having an activity companion facilitate children’s physical activity [27]. Schools directly increase children’s physical activity through physical education classes, extra-curricular sports, and exercise breaks [28]. Schools and after-school programs also provide time outdoors, which increases physical activity and decreases sedentary behaviour [39]. Since school day activities are similar for most children (e.g., a class has recess or physical education together), it is after school hours that differentiate children who are inactive from those meeting physical activity guidelines [40]. Most children with CCHD reported going home after school, where activity was primarily inactive (e.g., screen time). Those attending after-school programs reported some structured physical activity. Since children with CCHD are much less likely to attend after-school programs [41], their inactive after-school lifestyles may reflect reduced opportunities for physically active peer play. Intervening to engage these children in after-school physical activity programs may significantly impact their participation.

Contrary to earlier healthcare narratives regarding overprotection [26], the results from this study suggest that parents of children with CCHD do not “bubble wrap” their children into inactive lifestyles. All parents firmly believed that physical activity was very important and they portrayed their children as leading active lifestyles, despite objective measures to the contrary [15]. Overestimates of children’s physical activity occur among 80% of parents, whether children are healthy [42] or have CCHD [22]. Enabling more accurate perceptions of their children’s lifestyles may increase critically important parental physical activity support. Current research is assessing the impact of sharing objective physical activity data with children with CCHD and their families [43].

Being physically active while living with CCHD presents unique challenges. Children typically display one of two patterns of behaviour in the face of challenging situations [44]. Some children respond by persevering despite failure, making positive-affective statements and maintaining expectations for future success (mastery pattern). Other children respond to challenges with deteriorating performance, negative affect and low expectations for future success (helpless pattern). The responses of children in this study indicated that they predominantly followed the helpless pattern of response in relation to physical activity participation as they anticipated limited success (“I can’t keep up”) or injury potential (“What if I get hit in the chest?”). How parents or significant others (e.g., teachers) respond and support the child who is facing a challenging situation can shape how children respond [45]. Encouraging persistence, task-focused teaching and high positive affect will support mastery patterns of behaviour. Focusing on the outcome (i.e., failure), encouraging the child to change activities, and negative statements about the child’s ability support helpless behaviour patterns [44,45]. It is unlikely that adults (parents, teachers, etc.) who are uncertain about a child’s physical activity capacity could respond to attempts to be active in a manner that would support a mastery pattern of behaviour. Parents of children with CCHD also have a high incidence of mental distress (30% to 80%) with 40% relying on psychiatric care [46]. Such health concerns would be expected to further limit their ability to positively support children in overcoming challenging situations, such as those encountered when trying to be active with peers. Finally, longitudinal research indicates that parent attachment styles impact their ability to respond to the needs of their children with CCHD [47]. Parents who use an avoidance strategy strive to maintain behavioural independence and emotional distance. Such strategies enable effective coping when stressors are relatively minor (e.g., simple heart defects), but are insufficient and become overwhelming under situations of significant distress (i.e., CCHD). Avoidant attachment behaviours at the time of CCHD diagnosis have been associated with lower children’s self-concept at 7 years of age, indicating the long-term impacts of limited parent coping strategies [47]. Research is required to identify effective interventions that will better enable healthcare providers, parents of children with CCHD and other significant adults (e.g., teachers) to support mastery patterns of behaviour related to childhood physical activity.

Parents, and their children with CCHD, reported physical activity uncertainty as a dominant discourse in the home and health environments. Children’s perceived physical activity self-efficacy and expectations for success are well-established correlates of increased participation [36]. Parents commented on their “balancing act” and “parenting moments” when making decisions about appropriate activities for their children, reflecting similar concerns expressed by parents of adolescents with CCHD [20]. The results of this study extend the knowledge of how interactions that lead to uncertainty are interpreted as discouraging physical activity even for younger children. For children, uncertainty would be expected to reduce activity self-efficacy. Parents would be expected to “err on the side of caution” in situations they interpret as potentially threatening to their children’s health or safety. There is a clear need to understand how the CCHD health environment can reduce parent and child uncertainty about physical activity.

Parents, but not children, clearly reported the physical activity guidance provided by the children’s cardiologists and none indicated that they received equivocal advice. Parent clarity about the advice received contrasts with reports of discrepancies between parent and physician reports of physical activity restrictions for children with CCHD [23]. Parents and children expressed concern about the child being hit in the chest, recollections likely stemming from the children’s post-surgical recovery when they would have been instructed to protect the healing sternum from impact. These findings suggest that discrepancies between parent and physician-reported activity restrictions may reflect different recall timeframes. Physicians discuss the restrictions currently applicable to the child. These study results suggest that parents accumulate the guidance received over the life of their child, adding rather than replacing previous advice when newer comments are received. The guidance accumulated over time would tend to generate uncertainty as it would change as children grow and their CCHD is treated or evolves.

Trying to reconcile physician guidance with questions about their children’s daily activity also generated uncertainty. Is soccer or t-ball a “contact sport”? Why is soccer okay in gym class but not in a house league? Should my child sit out of gym class when the children are divided into teams for a “competition”? What if the ball hits him in the chest? My child makes everything a competition. What does no competitive sports mean? Should you push a child who is never active if the doctor said she should self-regulate her activity? Although parents felt that physician guidance was clear, many questions arose as they tried to implement that guidance into daily life. These results confirm and extend previous research suggesting minimal cardiologist physical activity restrictions, intended to minimize preconceived limits, often leave parents and children feeling uncertain about what activities are safe and appropriate [25]. Parental uncertainty is particularly important because it is strongly associated with children’s active lifestyles at this age [36,48]. Previous reports have identified the importance of uncertainty for the physical activity of adolescents with CCHD [25]. Parents reported that ambiguity or a lack of specific activity advice led their adolescent children to “limit themselves”. This study provides novel evidence regarding the impact of uncertainty among younger children, suggesting that health environment interactions need to help patients interpret physical activity in relation to their CCHD long before adolescence. To address feelings of uncertainty, discussions with children with CCHDs and their parents about current physical activity capacity should be prioritized within the health environment [49]. Such discussions should specify what the child can do now and how current recommendations may differ from previous advice. Future intervention research should target parent and child uncertainty so that home and health environments unequivocally encourage a physically active lifestyle for children with CCHD.

### Strengths and Limitations

This interview/focus group study had a robust sample size, beyond that required to identify both major and more variable concerns [50]. Data saturation was achieved. Participants agreed to enroll in physical activity research, which may have predisposed them to view physical activity positively. Whether the perceptions of children and parents who chose not to participate would differ is unknown. Interviews were conducted at the beginning of a physical activity intervention study. Member checking of results was not completed to avoid potential impacts on the longitudinal study results.

## 5. Conclusions

Children with CCHD and their parents recognize the importance of physical activity and having fun with friends. Uncertainty within the home and health environments regarding physical activity capacity contributes significantly to the inactive lifestyles of these children, despite minimal restrictions from health professionals. Research to enable parents, teachers and others in the community to distinguish between the physical activity limits imposed by the CCHD and other reasons that may discourage children with CCHD from being active (e.g., lack of interest, concern about “keeping up”) would enable these important adults to consistently and confidently support healthy active lifestyles among these children. Physicians and allied healthcare professionals can help patients and families overcome their uncertainty by educating both parents and children and encouraging the specific types of physical activity that are appropriate to each child’s heart condition. It is also important to discuss each child’s heart condition and treatment history in relation to how physical activity expectations and restrictions may have changed with treatment or over time.

Further research is recommended regarding the role of peer environments, such as school and daycare, in promoting healthy active lifestyles among children with CCHD. Having fun with friends is a key facilitator and “not being able to keep up with peers” is an important barrier. Research to identify leader roles/skills and environmental designs that lead to “fun” and “keeping up” with peers may provide more positive support for physical activity among children with CCHD. Research is also required in order to create health and home environments that better support the physically active lifestyles associated with optimal health and higher childhood quality of life.

## Figures and Tables

**Table 1:** Demographic Description of Participants Completing a Focus Group Only.

	Mother A	Father B	Mother C	Mother D & Father D	Mother E
Child Sex	Female	Female	Male	Male	Male (non-participant)
Child Age	11.5 years	9.8 years	10.5 years	8.6 years	4.9 years
Child Diagnosis	Tetralogy of Fallot	Pulmonary Atresia, Hypoplastic Tricuspid Valve	Ventricular Septal Defect, Coarctation of Aorta	Aortic Stenosis	Hypoplastic Left Heart Syndrome
Child Surgical History	Biventricular Tetralogy Repair	Fontan	Coarctation & VSD repair	Valvotomy	Fontan
Pacemaker	No	Yes	Yes	No	No

**Table 2:** Demographic Description of Interview Participants.

Child	Sex	Age	Diagnosis	Years from Fontan	Pacemaker	Parent
1	M	9.6	DORV	5.5	No	Father
2 ^F^	M	10.5	HLHS	1.4	No	Both GP
3 ^F^	M	10.8	DILV	4.4	No	Father
4	M	8.6	HLHS	6.3	No	Mother
5	M	7.2	DILV	4.7	No	Both
6	M	9.0	Tri. Atresia	6.8	No	Mother
7	M	10.4	DORV	7.1	No	Mother
8	M	7.1	Tri. Atresia	4.4	Yes	Mother
9	M	8.3	DORV	3.8	No	Mother
10	M	11.2	DILV	6.6	No	Both
11	M	6.2	Tri. Atresia	4.2	No	Mother
12	M	6.4	HLHS	3.3	No	Mother
13	M	8.2	HLHS	5.9	No	Mother
15	M	7.8	Tri. Atresia	5.2	No	Mother
16	M	6.4	Pul. Atresia	2.5	No	Both
19 ^F^	M	11.6	HLHS	9.1	No	Both GP
20	M	8.3	HLHS	5.1	No	Mother
25 ^F^	M	11.2	HLHS	8.9	No	Mother
27	M	9.7	HLHS	3.4	No	Mother
30 ^F^	M	9.1	HLHS	5.3	No	Father
33 ^F^	M	7.9	Tri. Atresia	5.0	No	Mother
201	M	11.4	Tri. Atresia	9.2	No	Both
202	M	9.7	DILV	6.8	No	Both
203	M	7.9	Tri. Atresia	5.2	No	Mother
1 ^F^	F	10.5	DORV	7.7	No	Both
2	F	7.7	DILV	4.8	No	Both
3	F	7.1	DILV	3.3	No	Both
4 ^F^	F	7.0	HLHS	2.6	No	Mother
5	F	6.0	DILV	3.1	No	Mother
6	F	10.6	DORV	4.4	No	Mother
7	F	6.7	Tri. Atresia	4.0	No	Both
8	F	11.1	DORV	9.6	No	Father
9 ^F^	F	6.1	DORV	1.0	Yes	Mother
10	F	10.2	Tri. Atresia	7.6	No	Mother
11	F	9.7	DORV	7.5	No	Father
17	F	10.5	Pul. Atresia	7.4	No	Mother
18 ^F^	F	6.4	DILV	3.4	No	Grandmother
19 ^F^	F	9.1	DILV	7.2	No	Both
20	F	9.2	DORV	7.3	No	Mother
23	F	9.9	DILV	7.0	No	Mother
24	F	10.9	DILV	8.9	No	Mother

^F^ = Participant in interview and focus group; DORV = double outlet right ventricle; HLHS = hypoplastic left heart syndrome; Tri. Atresia = tricuspid atresia; Pul. Atresia = pulmonary atresia; DILV = double inlet left ventricle; GP = grandparent.

## Data Availability

To protect the privacy of the study participants, de-identified study data are available from the corresponding author upon request.
